# Reports of Cities, Towns, and Districts.—Bombay

**Published:** 1859-01

**Authors:** 


### SANITARY AND SOCIAL SCIENCE.

### REPORTS OF CITIES, TOWNS, AND DISTRICTS.

### SANITARY CONDITION OF BOMBAY.

"Our knowledge of the real sanitary state of Bombay is vague and unsatisfactory. It is quite true that we have weekly, monthly, and annual mortuary reports, of the most elaborate kind, but these at present do little more than tell us of the fluctuations in the *mortality* of the people. They give us no means of comparison with any other place, simply because we have no correct knowledge of the population. In fact, only a short time ago we saw in the *Bombay Gazette* an attempt made to estimate the population, from data that must ever be looked upon as a compound of error. Speaking of the mortuary return for 1857, the writer says:—

"'It will be seen that the total number of deaths from all causes amounted to 18,162, against 15,069 in the preceding year, being an increase of 3,093, or about twenty per cent. The increase of mortality is shown to be amongst all classes of the community, arising as well from natural causes as from epidemical disease. Owing to the undefined estimates generally formed of the population of this place, it is next to impossible to calculate with anything like certainty the rates of mortality here as compared with other places. It is shown by the calculations and returns of the Registrar General, that in England nine persons and a half in every thousand die annually. Supposing that to be the rate of mortality in Bombay, the population would amount to 1,901,294; but calculating the deaths at thirty per thousand, or three per cent., the population would number 605,400, which may perhaps be some approximation to the truth. For a town like this, situated in a tropical climate, badly drained, imperfectly cleansed, and the houses generally without proper ventilation, the public health does not appear to suffer so much as might be anticipated.'

"In the first place, we should like to see *where* the Registrar General's calculations and returns show a mortality in England of nine and a half per thousand. In such a case the span of life in England, instead of averaging about forty years, would be far above seventy, and we know that no-one supposes it is so high as that.

"For Calcutta we find the following statistics in the *Friend of India* of 1st July, 1858:—

"'According to the last Conservancy report, the number of deaths during the past year in Calcutta was 18,504 persons. Of these 883 were Christians, 13,266 Hindoos, and 4,425 Mahomedans. The Commissioners state that of the European inhabitants 6·20 per cent. die every year, of the Hindoos 4·83, and of the Mahomedans 3·99. The *Hindoo Patriot* remarks on this immense discrepancy between European and Native mortality, and accounts for it by the fact that Natives when ill or old leave Calcutta and go to the Upper Provinces. There is another disturbing cause —one-third of the Hindoos and Mahomedans in Calcutta are unmarried, and the returns therefore do not include many children.'

"Calculating from these data, we may express the population of Calcutta as 399,801, and the average mortality for last year as 4·62 per cent. This is in round numbers about double the average mortality in England (2·27), and France (2·5), and we believe that the mortality in Bombay will, when we have data to calculate it, be found to be not very much below that of Calcutta, supposing that we have then a more trustworthy guide to the population of Bombay than that suggested by the Chief Commissioner of Police. We must put aside his last few lines of deductions in reference to the public health not suffering 'so much as might be anticipated' as founded on premises altogether erroneous, and thus not entitled to consideration.

"We know from the mortuary returns that the average number of deaths in Bombay during the last nine years has been 15,023 per annum, and we find that during the past year the number has increased to 18,162; we know that the mortality among sepoys in Bombay is 24·66 per thousand—more than double that (11·9) of the Native Army of the Bombay Presidency generally; we know from actuarial returns of the value of human life that this mortality among the sepoys occurs at ages in which the expectation of life is favourable: we know that the average duration of life in Bombay varies from 23·5 years to 23·7 years; and considering these *facts* can we for one moment believe that 'the public health does not appear to suffer so much as might be anticipated?' We are not now dealing with one supposition—we quote or mention the sources from which we have derived our data, and we believe the facts will startle most men, as much as they did ourselves on completing our calculations. Indeed we find that in the only long calculation into which we feared error might have crept, our results are borne out by Dr. Leith's, that the average duration of life in Bombay is 23·5 years.

"We might enlarge upon the state of the population of Bombay to be taken into consideration with reference to the average age, viz.: that the immigration to it is very great, and that the greatest number of immigrants have passed the most fatal period of life. What the *Hindoo Patriot* alleges as to the proportion of unmarried in Calcutta, we may also surmise in an increased ratio with respect to Bombay, for we find that the births in Bombay are only about half the deaths. We insert a calculation of the different average ages of the deceased of the various castes in Bombay:—

| | | | |
|---|---|---|---|
| Buddhists and Jains | 25·3 | years. | |
| Brahmins | 27·9 | " | |
| Lingaets and Bhateeas | 25·9 | " | |
| Hindoos of other castes | 20·77 | " | |
| Hindoo Outcastes | 21·09 | " | |
| Mussulmans | 27·45 | " | |
| Parsees | 30·40 | " | |
| Jews | 36·10 | " | |
| Native Christians | 30·59 | " | |
| Indo-Europeans | 20·60 | " | |
| Europeans | 26·80 | " | |
| Negro Africans | 27·0 | " | |
| Unknown | 17·0 | " | average 23·7 |

"We are quite aware that this one year's table is of comparatively little statistical value, but it would be most useful to continue it for a period of ten years. The series of tables would then have not only a sanitary value, but also it would be a not unimportant contribution to Physiology.

"So much for our present statistics of population. We require an accurate census of Bombay, and of the various districts into which it is divided. We should wish also a street census. If we had these, and a repetition of the census four or five years hence, we should be able at once to construct maps shaded roughly, which would show at a glance the sanitary state, and the relation between overcrowding and mortality.

"The annual Mortuary Reports of Bombay furnish us with as full particulars of the deaths as we can expect at the present time. We must remember how few are the qualified practitioners of medicine, especially among the classes who furnish the greatest numbers of deaths. In 1856 we find that—

| | | | |
|---|---|---|---|
| 2,151 | persons died from | Cholera |
| 199 | ditto | Small-pox |
| 6,397 | ditto | Fever |
| 8,747 | | |

"We have omitted all the deaths from fever in which any complication or accompanying disease is named. We presume that the fever here named is of the intermittent type, though fever intermittent is afterwards credited with only two deaths. We are quite aware how difficult it is to learn the true cause of death from natives, but it does seem to us that the complications afterwards separately mentioned might be classed under the head of the disease that produced death. But we also find that distinctions apparently without a difference have crept in. Of this kind are 'Fever and Cough,' and 'Fever and Respiratory Disease,' again, 'Fever and Bowel Complaint,' and 'Fever and Diarrhœa.' We think that any medical man would be puzzled to assign distinctive characters to such complications as those above named. The returns are most voluminous, and evidence how much care and labour must have been bestowed upon materials that at the present time are not altogether trustworthy.

"The Report at the commencement of each year's return is most valuable, and gives the comparative mortality in different parts of the island as well as can be expected so long as we are without the much-to-be-desired census.

"The deaths from fever, cholera, and smallpox, and many of the diseases of the alimentary canal, must be looked upon as preventable, and this amount is far more than sufficient to make us deem any expense slight that shall reduce their frightful number. What is the estimated value of *mere* HUMAN LIFE? We are persuaded that none can answer this in a manner that would be deemed satisfactory by a dispassionate observer; but we think that if we simply state that on the most eminent authority several thousands of lives are *unnecessarily* sacrificed in Bombay by the demon of pestilence, we must effectually arouse attention to the subject. We know, however, that the subject in all its importance has received the careful attention of Government, and believe it is mainly a want of knowledge as to the necessary remedies that is now the stumbling-block to sanitary measures. We have already mentioned the care which is bestowed on sanitary measures by the two nations most advanced in civilisation in Europe, and recent Acts of the Legislative Council of India lead us to hope that a *measure* at least of the same care will be bestowed upon India. We believe that the first Sanitary Commissioner was appointed in Lucknow at the commencement of the memorable siege; what good he did, though crippled by want of labourers, we learn from the mention of his name in the narrative of the siege by General Inglis, and the occasional mention, though grudging and sarcastic, by Mr. Rees. We trust that we shall at some future period have his narrative of sanitary work, and also a history of the medical aspect of the 'Residency,' from first to last. We are sure that something of importance might be gained to sanitary science by such accounts.

"We find that besides the Government of Bombay, the more enlightened among the inhabitants are taking an increasing interest in questions relative to health. A petition, dated 21st May, to the Bench of Justices from a portion of the native community, shows that some at least are awakening to their true interests. The petitioners point out most serious defects in the administration of the Conservancy Act of 1856, and they state broadly that a large amount of bribery goes on amongst the inferior officers of the department in whose hands the cleansing of Bombay is placed. Upwards of six months ago we were told that such bribery as is described in the petition was common, more especially in respect to the dealing out of summonses to the police office for permitting accumulations of filth. We had no means of verifying those statements, but now that they have been publicly brought to the notice of the Justices of the Peace, we have no hesitation in alluding to them, and saying how firmly we believe that all are quite true. From the tenor of this petition we gather that at least a portion of the more influential members of the native community are convinced that the strict enforcement of this Act is really for their own good.

"We shall dismiss our next topic, water supply, with a very few words. We hope much from the spirit in which the Vehar Water Works are carried out, and that it is only the commencement of an entirely new state of matters in Bombay. Pipes carrying water should be introduced into every house in the densely populated parts of Bombay. Through the agency of this water supply and a new system of drainage, we hope at no distant day to see a greatly diminished number of deaths figuring in the mortuary returns, and consequently a more healthy town.

"At the present time the drainage of Bombay, both as respects the ordinary house drainage and the monsoon water, is as defective as can be. Even close to the most public roads we see vile attempts at drainage, that would disgrace a nation just emerging from barbarism. As an instance of this, the drain between Bellasis-road and the Dhurrumsala is sufficiently public to be more or less under the eyes of all. We shall describe what we saw going on for several months during the past year. That side of the Dhurrumsala fronting Bellasis-road has several small openings in it to permit sewage to pass to the monsoon water-way. Only one of these apertures is used as a drain, but immediately outside of this, as there is no artificial channel for the escape of the sewage to the water-way, a natural cesspool has of course formed. This is from six to eight feet in diameter, and consists of black filth exhaling a most noisome smell. After passing through this cesspool, the fluid formerly pursued a zigzag course until it was all absorbed by the ground in the driest weather, or a small portion might reach the main drain. Some months ago workmen were seen at this place, and we did think that some efforts were being made to do away with the nuisance. We watched the proceedings of these men with some curiosity, and we found that the plan adopted was something like the following. The workmen took earth from the sides of the channel, and *with great care* covered up the nasty looking cesspool, and the stream from it. This was allowed to dry (the supply in the meantime being stopped), and then a straight trench was dug in the place of the former little zigzag. This was judged sufficient, but of course as soon as the supply of sewage recommenced to flow, the cesspool and drain returned to their former state. The mode of cleansing this open ditch was quite as primitive and effectual as the plan of repairing it. Occasionally early in the morning sweepers came and passed their brooms along the bottom of the ditch, until a supply of the black stuff was accumulated. This they took out and placed alongside the ditch to dry in the sun! We have seen the cleansing process repeated several times. We know not whose special duty it was to see after this ditch, but comment is needless. We have not exaggerated one iota. It may to some seem trifling to state a matter so unimportant, but a feather shows the direction of the wind, and this little trifling fact spoke volumes as to the system which allowed such a plan to be carried into execution.

"The drains, in the more densely crowded part of Bombay, are the worst that can be imagined—broad and uneven at their base, they usually course along immediately alongside the foundations of the houses, and stagnant pools of water are constantly met with. In every part the drains are far too large for the amount of fluid which they have to carry, hence of necessity the deposition of any matter that may be held in suspension takes place, the drain becomes blocked up, and the nuisance is occasioned. How can it be expected that houses should be healthy with their foundations saturated with sewage water, or with the constant exhalations from matter decomposing under the influence of a tropical sun? In some parts of Bombay we find drains made of chunam, open, of a square form, and occasional efforts made to keep them clean. These are attempts at improvement, but are faulty as to material and form, and in being uncovered. The material is faulty, for it is absorbent; the form is bad, for it is calculated to favour deposition; and not having any covering to keep in the gases, and to prevent the sun's rays acting upon both the fluid and solid portions, allows noxious gases to spread disease. If the chunam drains are thus spoken of, how can we characterise the gullies which separate many of the better native houses in the principal streets of Bombay? Let any mere superficial observer cast his eye on either side of the Kalbadavie-road, and glance at the gullies between the houses there, he will then get a clear notion of the filthy state of these apologies for drainage. The windows open into the upper part of these gullies, and we may easily imagine to what kind of atmosphere these windows will give entrance. The street doors of many of these gullies are with great prudence kept locked, but we venture to say that unless the state of affairs has vastly changed for the better within the last three months, any looker-on would see nothing in the open gullies except *degrees* of filth.

"If the real observer leaves his carriage and penetrates into the back streets, the degree of filth gets worse as he proceeds. We confess that in several of our peregrinations we were perfectly convinced that in some places the abortive attempts at drainage had done far more harm than good. The filth was quite stagnant, and being constantly kept moist by accessions of fluid matter, gave out the most disgusting effluvia; it seemed to us that it was only the constant residence in such an asmosphere that could render it endurable. Some months ago, an engineer of high standing remarked to us on some of the houses occupied by employés of Government in the Fort, that he was surprised, not at the men being sick, *but that they could live at all.*

"The main drain of Bombay, like the Fleet Ditch, has proved quite a failure, and we must, we conceive, put it down as irremediable. The old plan of drainage by cesspools can now only be mentioned to be scouted.

"Enough has been said to arouse men who wish to benefit their fellow-creatures. We believe that the evils which we have enumerated are remediable, or we had not thus laid bare some little of what we have seen of the back *slums* of Bombay. We know that the remedy (glazed tile drainage) has difficulties, but none so great as might not easily be surmounted by the engineers of an age that can make railways up the Bhore Ghaut, and carry railways in bridges across the Menai Straits.

"It is quite true that the drainage of London is at present an unsolved problem, but the difficulties that we meet with in London are three principally. 1st. The desire to keep the Thames unpolluted; 2nd. The distance of any other outlet for the sewage; and 3rd. The desire to utilise the sewage of London. Not one of these difficulties meet us in attempting the drainage of Bombay, the sea on almost every side does away with the first and second named, and the third named would surely excite a laugh if any one considered it as an objection to using the sea.

"For Bombay we believe it to be essentially necessary that there should be a foul water drainage system and a simple water drainage system. How far these may be combined, practical engineers must determine; it is essentially necessary to health that both foul and simple water be removed.

"At the present time we believe there is no survey of Bombay sufficiently accurate for the purpose of planning a complete system of drainage. Such a survey is urgently needed. For the perfect drainage of Bombay, several districts should have separate drainage systems, and each be discharged into the most appropriate and convenient part of the sea, beyond low water mark. It is probable that only a part of the town would require to be drained by the lifting system, but whether that part be small or great, or even the whole, we consider that a perfect system of drainage is absolutely necessary.

"As yet we have only spoken of the foul water drainage; simple water drainage is also indispensable to lessen the mortality which results from that tropical plague, intermittent fever. Not one patch of marshy ground should be left to generate the hitherto imponderable miasm only recognised by its effects. Marshes formed by simple water produce intermittent fever and its congeners, and by repeated attacks of these induce debility and organic disease. Want of drainage of foul water and filth, localise and spread epidemic diseases, and cause in the inhabitants who live near them a proclivity to epidemic diseases and general adynamic state of constitution, or in more familiar terms, a state of health below par.

"We have shown what plan is, we believe, required for the perfect drainage of Bombay. Even with great activity among the 'powers that be,' it would be years before any such comprehensive scheme as we have advocated could be carried out; but it must not be for one moment supposed that for the present we should be less active in making Bombay as clean as present circumstances admit. We should fine rigorously every householder whose drain or gully is not kept clean, and moreover we should make every householder responsible for the portion of street immediately in front of his premises being in a proper state. Let it be seen that it is the householder who is responsible for these matters, and in time *he* will take care that those, whose duty it is to keep the gullies, drains, and streets clean, at least do their work in such a way that he shall not be required at the police office.

"In every quarter of Bombay, public latrines should be established, and fitted with modifications of the apparatus recommended by the Board of Health. Public washing places for the dhobees should also be established, on such a plan that no difficulty may be found in conveying away the soiled water to the drains. With an abundant supply of water and good drainage, proper bathing places in the different sections of Bombay might be supplied at a very small cost.

"With the subject of drainage, we include several of the trades injurious to health which are carried on in Bombay. The most objectionable of these are the places where animals are kept, tanneries, slaughter and dyeing houses. Of the first named of these, the places appropriated to cows and bullocks are usually in a most filthy state, and must be regarded as detrimental to the public health. At every period of the year they are more or less of a nuisance, usually mere sheds, unpaved, and the liquid and solid manure are allowed to spread over the adjacent ground just as the laws of gravitation and absorption dictate, without assistance or guidance from man. The effluvia exhaled are constant, but during and after the monsoon much of the ground around these places become lakes of black liquid filth, which must exercise a most pernicious influence on the health of all living within the circle of their influence. We should like to see the opinion of the medical officers of a large native hospital as to the effect of these cow-houses or yards. In densely populated neighbourhoods no milch cattle should be allowed to be kept, as the mere contamination of the air by the breathing of a large number of animals, must have in the close nights of the hot season of Bombay a most baneful influence. Sometimes milch cows and buffaloes are kept immediately beneath dwellings. In addition to all human sanitary considerations, we have no hesitation in stating that the health of animals so placed must suffer, and if the animals themselves are in a bad state, it follows naturally that their milk cannot be of the same nourishing quality as that from healthy kine. We have not the smallest doubt that health and economy would be alike consulted by placing the whole of the milch cattle a short distance from Bombay. The manufacture of *fuel* should be strictly prohibited.

"The same remarks apply as forcibly to the places used as stables for the hack horses and bullocks of Bombay. Nowhere are thorough drainage and ventilation more urgently needed than in these miscalled stables. They are constantly placed on the ground floor of houses, and are not unfrequently only open at one end, one wall being deficient for light, air, ventilation, and every other purpose. Attempts are made at drainage in some of these places, but these are made with the worst form of drains, and they are constantly choked up.

"Many of the tanyards are in a dirty state, and all require drainage. The places where the skins are cleaned, and the hair removed, should be paved. A good supply of water is necessary.

"All indigo dye-houses should be placed at a considerable distance from dwelling houses.

"The proprietors of the slaughter-houses, in the present state of the drainage, should not allow the blood and other fluid offal to flow into the drains. Blood and other animal matter, in imperfect drains, tend to clog them up and to give off a large quantity of most offensive gas. They should be well supplied with water, and the utmost cleanliness should be insisted on. Lime-washing the walls every three months should be imperative.

"Of deficient house ventilation in Bombay as a cause of disease, we have little knowledge. As a general rule, we believe that it does not obtain to so frightful an extent as is met with in the poorer portions of towns in temperate climates. This is doubtless owing to the external temperature being so high, and thus it does not require to be excluded. The attention of the authorities in Bombay is, we think, much required to regulate the sheds and dhurrumsalas, where large numbers of itinerant mendicants and others lodge. These places are generally in a most unsatisfactory state, and though we have no means of verifying our supposition, we firmly believe that epidemic disease is not unfrequently rife among those who use them, and that these lodging-places serve as foci whence these diseases originate, or where at least they become localised.

"We note with great satisfaction the proposal lately made to provide European workmen with more suitable abodes, and we would ask, are there no rich natives with sufficient appreciation of the subject of proper habitations for the labouring poor of Bombay to expend a few thousands of rupees in providing, at different parts of the island, model houses, well supplied with water and drainage, and built on the most approved and economic plan, to show to the poor how much it is for their advantage in every way to live in well-arranged buildings? We believe that a few such sets of houses scattered here and there would do far more than years of other instruction to remodel the houses that are now dirty, ill-constructed, and unhealthy. To dignify this work, we would tell them that Prince Albert some years ago devoted much attention to this subject, and did much good. We are sure that there are numbers of scientific and practical men among the Engineers and Medicos who would gladly give their assistance. We know also that there are some natives of more enlightenment than their fellows who would point out what are the necessities for native dwellings of the class to which allusion is made.

"It was not originally our intention to discuss even cursorily any disease, or class of diseases, for we believe that the public perceive that certain diseases are preventable, but the recent epidemic of small-pox, and the discussions that have taken place in respect to the prophylactic power of vaccination, induce us to make a few remarks on small-pox. We consider the prophylactic power of vaccination as effectual in India as it is in European countries. We have seen two epidemics of small-pox in India, and cases occurring sporadically; we had a considerable number of cases to treat in the epidemics referred to, and we have seen more cases of small-pox occur in the same subject for the second time, than among those who bear the mark of successful vaccination. We have not treated a single case bearing the vaccine mark which was not markedly modified, and passed through the disease without any dangerous symptom. Doubtless the protective power of vaccination, as well as that of an attack of small-pox itself, wears out, and leaves the individual more or less amenable to a fresh attack. This is evidence[illegible] by the induction of the vaccine disease several times during the ordinary period of life. The same is true of an individual who has passed through an attack of small-pox,—he may in after years have a second, and some cases have been stated to have had a third attack.

"We repeat what we have before said, that we believe there is no difference in the protective power of vaccination, where it is properly performed, in Western India and in other countries. We are strongly of opinion that in Bombay the occurrence of epidemics of small-pox may be prevented by proper vaccine measures. Crowded dwellings in unvaccinated neighbourhoods are certain to be decimated by a disease whose contagious and infectious properties are greater than those of any other. We think that the vaccination machinery at present in operation in Bombay is as *inoperative* as it is possible to conceive. Bombay requires a vaccinator full of energy, and who has no other call upon his time. For years it will be no light work, the mere superintendence of vaccination in Bombay, if it is to be done effectually. We shall hereafter consider whether the office of vaccinator might not be placed in connexion with that of an office which we regard as absolutely necessary for Bombay.

"Of extramural interment little need be said. Its necessity has been recognised, and the Municipal authorities have only to see that no step backward is permitted.

"We have gone over, as we proposed, some of the more important defects in the sanitary condition of Bombay, and have treated them as bearing so directly and perniciously on the bodily constitution of those living within their influence. No one can doubt that these defects in sanitary police form the hot-beds in which the germs of epidemic diseases are located, and disseminated all around, producing as their fruit the deaths which we have designated as preventable. We have remarked upon some of the remedies, but, before going more particularly into the plans which we propose for ameliorating the condition of the state of things described, we must glance for one moment at the subject as bearing on man's superior part—the mind and moral development.

"If we look at this historically, we shall find that attention to sanitary matters proceeds with civilisation, but not with equal pace. Generally in country places cleanliness both of person and habitation is more attended to than in towns, but it is only from the difficulty and expenses that attend them in the latter places; hence epidemics arise to warn the inhabitants that their inattention to these matters is reacting. But in what parts of towns do epidemics arise, and from whence spread around?—In the most crowded, demoralised, poorest, and most ignorant quarters. Filth usually goes hand-in-hand with vice. Macaulay's graphic pages on the state of London in the time of the Stuarts illustrate this forcibly. Who that has visited much among the poor living in the purlieus of any large town in Great Britain has not the same experience to tell? It were needless and useless to attempt to settle, in such a case, which is the cause and which the effect, but that they are companions is as certain as possible. We believe that morality and cleanliness react on each other. What can be more demoralising than to live in a crowded part of a city whose atmosphere is tainted with foul emanations? What more depressing to the human mind than to see death from epidemic diseases decimating the people at your own door?

"We have not the least doubt that a well-arranged system of sanitary measures will materially assist the cause of education in Bombay.

"The appointment of a paid municipal council is a step in the right direction. We trust that the council will give a considerable amount of attention to the sanitary concerns of Bombay, but if it is anticipated that, among multifarious municipal concerns, the councillors will have time to attend personally to sanitary matters, we opine that such expectations will be grievously disappointed. It is possible that medical men may, among others, be chosen to sit at this Municipal Board, but sanitary legislation will only be one among many duties that the council will have to fulfil. It can hardly be expected with any show of reason that the councillors, or even one of their number, will be able to do anything in the way of an executive officer; and for real sanitary purposes we want men to see with their own eyes, hear with their own ears, and form their own judgments. We want men qualified by previous education and research to see what is required. We believe that men of sound common sense and business habits are the persons best fitted to be useful as Municipal Commissioners; they must at the same time have enlarged views, but above all decision and purpose of character. We should not wish to see men sitting at the Council Board who would on any occasion require one of King Alfred's 'Missi.' We cannot *as yet* look upon the Municipal Council as a great stride. It undoubtedly will take the place of the Board of Conservancy, and be well paid, but how it will do its work remains to be seen. We believe that an officer of public health is specially required: doubtless he should possess all the qualifications that we have enumerated for the councillor, but he must have in addition a knowledge of all that is known at the present day with respect to sanitary reform. He should be well paid,—his duties will be both very heavy and very responsible. His powers should be large. If only one officer of health is appointed, far more care should be lavished in the selection, and it would be necessary to entrust him with more power than if a board of health were to be established. The selection of a medical man for this appointment would be difficult, and a still greater difficulty arises as to the power with whom the appointment should rest. We should be very sorry to see one iota of personal interest entering into the selection. We have said that the salary should be large, we now say more,—that it should be sufficiently large to induce a man of some note and promise in the department to leave England and accept it.

"We have insisted on the necessity of a medical officer as Superintendent of Health in Bombay; we believe that one, however energetic, will be found totally unable to do the work. From the first we would suggest that he have two selected graduates of the Grant College as his assistants.

"The Officer of Health should be furnished with weekly reports by every medical practitioner, as to the deaths and sickness in each quarter of the town. This return should in the times of epidemics be furnished daily, and there would thus be a chance of combating the foe on the most favourable terms. The Registrar of Deaths and the Police authorities should also communicate constantly all particulars obtained with reference to disease and mortality that come to their knowledge. Each specified unhealthy locality should be visited as early as possible, and the Officer of Health should then and there endeavour to trace the origin of the disease, and if its cause is removable, an emergent requisition should at once be forwarded to the Superintendent of Repairs or other officer to be appointed by the Municipal Board. This "emergent requisition" at once raises the question, Is any requisition of the Officer of Health to be carried out without the sanction of the Municipal Council? If it is not, and the council sits but once a week, we may expect little good in the cutting short of epidemics by any sanitary means. It will be only a roundabout way of doing business if every requisition requires sanction. If the councillors take time for consideration, they may be much pressed with work, or the emergent requisition only coming before them in due routine, the hour for useful action will have passed in all probability, and little in the way of good be done. Our own belief is, that in such emergent matters, the requisition should be obligatory on the proper executive officer, and if he delay one hour unnecessarily, on his head be the responsibility. It is on such grounds as these that we have advocated the appointment of a sanitary officer of high standing. He must be not only a theoretic sanitarian, but also a practical one, and one who is willing and able to take responsibility; he ought to be in constant communication with the Municipal Council, and doubtless he will constantly consult and be consulted on sanitary matters. We are sure that an Officer of Health would have every support from the Magistrates, and summonses to the Police Office from him would do away with such a petition to the Bench of Justices as that we have commented on.

"We have before alluded to the connection that might be formed between the sanitary and vaccination offices. We believe that the offices might be joined. (We must not be misunderstood as wishing to give a plurality of offices.) Vaccination is essentially a branch of sanitary science, because the sanitary officer will have the places where vaccination is most wanted constantly brought before his eyes at the time when it will be most gratefully received. We believe that both sanitary and vaccination measures must be systematized, and we are not sure that it might not be a good arrangement to have two sanitary superintendents for different districts, to whom the charge of vaccination should also be committed. By this means constant supervision would be exercised over the subordinate vaccinators. No return should be sent in of which a very considerable degree of certainty did not exist. With some such arrangement, energetically carried out, we believe that if another epidemic of small-pox, such as has occurred during the present year in Bombay, did reappear, that it would be the last for many years; and moreover, were the offices united, that in a comparatively short time the vaccinator's staff would become an efficient secondary staff for vaccination purposes.

"In addition to the duties that we have hinted as appertaining to the Superintendent of Health, there is another to which we attach much importance, viz., the instruction of the educated portion of the natives in the general principles of health. Lectures once a week should be given at the Grant Medical College, and these lectures might be made both interesting and instructive to rising Bombay. The importance of the subject is everywhere recognised in Europe, and we feel sure that its importance has only to be pointed out in Bombay to have its full share of recognition."

[From a review entitled "*Sanitary Aspect of Bombay.*"]

